# Powerful Electron-Transfer Screen-Printed Platforms as Biosensing Tools: The Case of Uric Acid Biosensor

**DOI:** 10.3390/bios12010002

**Published:** 2021-12-21

**Authors:** Rocco Cancelliere, Alessio Di Tinno, Antonino Cataldo, Stefano Bellucci, Laura Micheli

**Affiliations:** 1 Department of Chemical Sciences and Technologies, University of Rome Tor Vergata, Via della Ricerca Scientifica 1, 00133 Roma, Italy; rocco.cancelliere@uniroma2.it (R.C.); alessio.ditinno@uniroma2.it (A.D.T.); 2 INFN-Laboratori Nazionali di Frascati, Via E. Fermi 54, 00044 Frascati, Italy; antonino.cataldo@lnf.infn.it

**Keywords:** nanomaterials, screen-printed electrodes, uric acid, point-of-care device

## Abstract

The use of carbon nanomaterials (CNMs) in sensors and biosensor realization is one of the hottest topics today in analytical chemistry. In this work, a comparative in-depth study, exploiting different nanomaterial (MWNT-CO_2_H, -NH_2_, -OH and GNP) modified screen-printed electrodes (SPEs), is reported. In particular, the sensitivity, the heterogeneous electron transfer constant (k^0^), and the peak-to-peak separation (ΔE) have been calculated and analyzed. After which, an electrochemical amperometric sensor capable of determining uric acid (UA), based on the nano-modified platforms previously characterized, is presented. The disposable UA biosensor, fabricated modifying working electrode (WE) with Prussian Blue (PB), carbon nanotubes, and uricase enzyme, showed remarkable analytical performances toward UA with high sensitivity (CO_2_H 418 μA μM^−1^ cm^−2^ and bare SPE-based biosensor, 33 μA μM^−1^ cm^−2^), low detection limits (CO_2_H 0.5 nM and bare SPE-based biosensors, 280 nM), and good repeatability (CO_2_H and bare SPE-based biosensors, 5% and 10%, respectively). Moreover, the reproducibility (RSD%) of these platforms in tests conducted for UA determination in buffer and urine samples results are equal to 6% and 15%, respectively. These results demonstrate that the nanoengineered electrode exhibited good selectivity and sensitivity toward UA even in the presence of interfering species, thus paving the way for its application in other bio-fluids such as simple point-of-care (POC) devices.

## 1. Introduction

Critical importance in electroanalytical measurement is given to the magnitude of the electrochemical response and its reproducibility. There are different ways in which this target can be achieved: first, improving the electron transfer conductivity of the transducers; second, reshaping the geometry of the working or reference electrode [1] (avoiding post-printing modification procedure); finally, attempting to alter the surface area of the electrode. Among these, the electronic transfer is the cardinal attribute behind all the different electrochemical sensors. High electronic transfer values ensure satisfactory efficiency and adequate electrochemical performances for all the different electrochemical platforms. In the field of electrochemistry, the parameter that fully describes the aforementioned process is the heterogeneous electron transfer constant (k^0^). This is strictly dependent on the material that makes up the electrode and gives the electrochemist an indication regarding the electron transfer rate between an electroactive species and the electrodic surface. Randles’ theory [2] and Marcus’ theory [3], respectively, are the most important theories that allow us to completely measure and understand this parameter. In particular, Randles’ work, developed in 1947, describes the determination of the heterogeneous electron transfer constant using electrochemical impedance spectroscopy; whereas, Marcus’ model, elaborated in 1956, reports the voltammetric determination of the k^0^. The extensive comprehension of this electrochemical feature, along with the growing demand for super-sensitive and reliable electrochemical sensors, have paved the way, over the last decade, for the development and applications of innovative technologies and materials. In this overall scenario, some works dealing with electrochemical sensors based on nanomaterials-modified platforms are frequently reported in the literature [4,5,6]. However, despite its crucial importance, only a few papers focus on the role of k^0^. Nanomaterials, objects having at least one nanometric dimension, have been extensively investigated due to their outstanding chemical, mechanical, magnetic, and electrical properties, given by their small-scale sizes. Moreover, the electrochemical field benefits from the enhanced electrochemical performances of these materials (e.g., sensitivity, stability, selectivity) applied in the modification of electrodic platforms.

Among all the viable nanomaterials, carbon-based (CNMs) have widely been used due to high conductivity, chemical stability, wide voltage range, and fast heterogeneous electron transfer properties, inherited by the carbon electrode ancestors and enhanced by the nanoscale effect. Carbon nanotubes and graphene reported significant findings in electrochemical fields [7,8,9,10]. Their electrochemical performances are related to their high conductivity due to their particular electronic structures. The extensive literature reflects their importance in the modification of electrodes: they were used to detect inorganic and organic analytes, decreasing the limit of detection [11,12,13,14]. In addition, not only do the “classic” sensors take advantage by using nanomaterials, but the biosensors have experimented with a new burst, too. In fact, the realization of the redox reaction driven by enzymes on pristine electrodes is complicated because the active centers of most biomolecules are located in a deeply positioned hydrophobic pocket [15,16]. It was demonstrated in [17] that carbon nanotubes enhanced the direct electron transfer capability between enzymes and electrodes; on the other hand, due to its extraordinary electron transport property and high specific surface area, graphene has promoted the electron transfer between electrode substrates and enzymes. Lastly, nanomaterials provide improved electrocatalytic activity and minimize the electrode surface fouling: these two features make their use extremely advantageous in biosensor development [6,17,18,19,20].

Most analytical devices were realized by means of screen-printed electrodes (SPEs) [18,21,22,23]. These platforms, whose proliferation during recent years has been extraordinarily important in bench and in loco analytical measurements, are obtained by printing different inks on various plastic substrates exploiting thick film technology. SPEs are generally made of three different electrodes (working electrode-WE, counter electrode-CE and reference electrode-RE) realized using various inks. The main goal in the analytical field of sensors is to develop sensitive, inexpensive, and user-friendly platforms [24,25], whereby these electrochemical devices are generally modified with various nanomaterials in order to improve their sensitivity, repeatability, and dependability. The main characteristic of a super-sensitive electrochemical platform is a fast electron transfer process, therefore an effective discharge of the analytical probe at the electrode–solution interface. To ascertain this capability, four fundamental parameters are usually studied: k_0_, ΔE, E°, and Ipa and Ipc, respectively [26]. Carbon-based inks, constituting the WE of SPEs, can be easily modified with electrochemical enhancers: carbon nanomaterials, such as carbon nanotubes (CNTs) or graphene, are among the most prominent examples of materials used for post-printing SPEs modification. The component atoms of graphite, graphene, or nanotubes share the same fundamental structural arrangement in which each structure is made up of six carbon atoms that are tightly bound together in the shape of a regular hexagonal lattice [27]. A singular graphene sheet can be wrapped into a zero-dimensional spherical fullerene, rolled into a one-dimensional carbon nanotube, or multiple graphene sheets can be stacked one upon each other into graphene-nanoplatelets (GNPs) [28]. This is well known in the literature as it confers an excellent electrical conductivity when applied in the modification of disposable screen-printed electrodes [29,30]. These nano-modified electrodes have been applied in the buildout of an enzymatic biosensor able to monitor and quantify uric acid (UA) in the nanomolar (nM) concentration range. UA is the ultimate catabolite of purine metabolism in humans and higher primates and plays a clinically valuable diagnostic role [31]. The extent of UA acid levels can be related to several diseases and conditions: gout, hyperuricemia, Lesch–Nyhan syndrome, hypertension, diabetes, kidney disease, and cardiovascular disease [32,33,34]. 

Many analytical methods have been developed for the determination of UA, ranging from simple colorimetric procedures to fluorimetric [35,36] and chemiluminescence (Chemlum.) methods [37,38], to those of flow injection [39] and high-resolution separation, such as capillary electrophoresis (CE) [40,41] or high-performance chromatography (HPLC) [42,43]. Although widespread, these methods usually lack the selectivity required for UA quantification in complex biological matrices: these difficulties are mainly due to the presence of interfering species. To overcome this problem, various kinds of electrochemical sensors and biosensors for UA determination have been implemented and tested as alternative methods to the aforementioned staple analytical techniques. A comparison between the most representative methods is reported in Table 1.

Among all the different proposed methods, the method based on the enzymatic reaction (*uricase*) shows up as the most robust. In these biosensors, the selective and sensitive recognition of UA is obtained using *Uricase*, which catalyzes the following reaction (Equation (1)):

(1) $$\mathrm{uric}\;\mathrm{acid}+O_{2}+H_{2}O\;\overset{\;\;\;\;\;Uricase\;\;\;\;\;}{\to }\;\mathrm{allantoin}+{\mathrm{CO}}_{2}+H_{2}O_{2}$$

The electrochemical oxidation of the produced H_2_O_2_ (Equation (2)) allows the amperometric detection of uric acid [32,50,51,52].

(2) $$H_{2}O_{2}\;\overset{\;\;\;\;\;\;\;\;\;}{\to }\;O_{2}+2H^{+}+2e^{-}$$

However, the employment of conventional electrodes (i.e., Pt, Au, carbon, etc.) in H_2_O_2_ oxidation can represent a serious problem due to UA oxidation at such positive potentials. The use of mediators, in our case Prussian Blue (PB), can help solve this problem. As demonstrated in our previous work [24], the deposition of PB on WE allows us to determine the concentration of H_2_O_2_ at a fixed potential (50 mV), exceeding the above-described problems.

This paper describes the morphological and electrochemical characterization of bare, nanomaterial-based SPEs obtained using unmodified and functionalized MWNTs and GNPs. Particular attention has been paid to the electron transfer process, constituting the fundamental process of the increased sensitivity of the nano-modified platforms. The aim of this paper is to properly modify the electrodic surface area by means of various carbonaceous nanomaterials, comparing the electrochemical outputs to determine the most convenient ones in terms of sensitivity and repeatability. In order to ascertain these properties, scanning electron microscopy (SEM), Raman spectroscopy, cyclic voltammetry (CV), and square wave voltammetry (SWV) analysis were performed in the presence of potassium hexacyanoferrate (III), obtaining important information about SPEs’ analytical performances. The electrochemical outputs deriving from nanoengineered electrodes have been compared to the performances of benchmark, bare SPEs. Hence, the purpose of this study was to develop a biosensor incorporating uricase immobilized onto CNMs-based PB-modified SPEs for the quantitative determination of UA, which is directly proportional to the produced H_2_O_2_. Encouraging results in terms of selectivity, sensitivity and reproducibility toward UA have been obtained, thus paving the way to their feasible application in other bio-fluids such as simple point-of-care (POC) devices.

## 2. Materials and Methods

### 2.1. Materials and Methods

All chemicals from commercial sources were of analytical grade. Ethanol, glutaraldehyde solution, potassium chloride, uricase (U0880-250UN), and uric acid were purchased from Sigma-Aldrich (Steinheim, Germany). Potassium ferricyanide was purchased from Fluka Chemie, Sigma-Aldrich (Buchs, Switzerland). Bare MWNTs, MWNT-OH and -CO_2_H, functionalized MWNTs were purchased from Heji Inc. (Hong Kong). –NH_2_ functionalized MWNTs were purchased from (Waltham, MA, USA). GNPs were produced by micro-cleavage exfoliation of the expanded graphite (provided by Asbury^®^, Wilmore, KY, USA), as reported in previous studies [53]. The buffer solution used is 0.05 M phosphate buffer saline (PBS), 0.1 M KCl, pH = 7.4.

### 2.2. Electrodes

Screen-printed electrodes (SPEs) were produced in-house with a 245 DEK (High-performance multi-purpose precision screen printer, Weymouth-UK) screen-printing machine. These devices are composed of three electrodes: a working (WE), a counter (CE), and a reference (RE) electrode, respectively. In particular, the WE (apparent geometric area of 0.07 cm^2^) and CE are deposited using a graphite-based ink (Elettrodag 421) from Acheson (Milan, Italy); whereas, the RE is produced using a silver ink (Acheson Elettrodag 4038 SS). The electrochemical cell (WE, CE, RE) is finely defined using an insulating layer (Argon Carbonflex 25.101S).

### 2.3. Apparatus

Cyclic voltammetry (CV), square wave voltammetry (SWV), and chronoamperometric analysis were performed using an Autolab electrochemical system (Eco Chemie, Utrecht, The Netherlands) equipped with PGSTAT-12 and GPES software (Eco Chemie, Utrecht, The Netherlands). Dispersions were realized using Hielscher UP200St-Ultrasonic Transducer. Morphological analyses were performed using a VEGA II scanning electron microscope (Tescan, Czech Republic). Raman spectra were performed using an Invia Raman microscope (Renishaw, UK) endowed with a 532 and 633 nm laser, a 100× objective, and an 1800 L/mm grating.

### 2.4. Preparation of MWNT or GNP-Modified SPEs

The carbon nanomaterials employed to modify SPEs have been prepared as reported below. Initially, screen-printed electrodes were amperometrically (1.7 V, 180 s) pre-treated using a 0.05 M phosphate buffer + 0.1 M KCl, pH 7. At this point, once rinsed using distilled water (to remove salt residues), the electrodes were modified using CNMs. The nanomaterials were prepared for dissolving using an ultrasonic transducer (200 W, 26 kHz and 30 min), 1 mg of each powder in a 2:1 ethanol–water mixture to a 1 mg mL^−1^ concentration. In particular, the drop casting procedure (6 μL of each CNMs dispersions) was employed to modify the WE of our screen-printed platforms. Once dried at room temperature, the modified CNMs were ready to use.

### 2.5. Preparation of Uricase-Based Biosensors

SPEs have been modified with Prussian Blue (PB). The procedure, here applied, was optimized in our previous work [24]. Subsequently, the PB-modified platforms were cast using CNMs as detailed in Section 2.4 Uricase immobilization on the WE surface was realized by exploiting the functional groups of the CNMs with the addition of glutaraldehyde (1% *v/v*). In particular, a 2 mg mL^−1^ *Uricase* solution (10 Umg^−1^, 0.1 U each electrode) was prepared using 0.015 M PBS pH 7.4. The biosensors were stored at 4 °C in a humid chamber.

### 2.6. Analytical Parameters Calculation

From the analysis of ten different blank samples (in the absence of the requested analyte), the standard deviation (SD) of the obtained current values was estimated. Thus, the limit of detection (LOD) was calculated using Equation (3):

(3) $$\mathrm{LOD}=\frac{3\cdot {\mathrm{SD}}_{\mathrm{blank}}}{S}$$

where SD_blank_ is equal to the standard deviation of the blank samples and S is the slope of the calibration curve.

The electronic transfer process was studied using the heterogeneous rate constants [54] (k^0^) for the redox process: [Fe(CN)_6_]^3−^ + 1 e^−^ ⇄ [Fe(CN)_6_]^4−^_,_ as described in detail in our previous work [55]. The k^0^ was calculated using Equation (4):

(4) $$k^{0}=\varphi \sqrt{\frac{D_{0}\pi \nu nF}{\mathrm{RT}}{\left(\frac{D_{R}}{D_{0}}\right)}^{\alpha }}$$

where D_0_ and D_R_ are the diffusion coefficient for the ferricyanide (D_0_) and ferrocyanide (D_R_), ν is the scan rate (vs^−1^), n is the number of electrons involved in the process, F is the Faraday constant (mol^−1^), T is the temperature (K), R is the universal gas constant (JK^−1^ mol^−1^), and α the dimensional transfer coefficient [56]. In particular, assuming the ratio of the anodic and cathodic peaks are approximately equal to 1 (Ipa/Ipc = 1), a dimensional transfer coefficient equal to 0.5 was chosen. 

According to the Nickolson method [54], where there is a correspondence between each ΔE and φ value, the parameter φ can be obtained using Equation (5) [57]:

(5) $$\varphi =\frac{\left(-0.6288+0.0021\cdot \Delta E\right)}{\left(1-0.0170\cdot \Delta E\right)}$$

The Randles–Sevcik equations (Equations (6) and (7)) were exploited for the diffusion coefficients and the percentage increase in faradic current estimation, respectively.

(6) $$I_{p}=\left(0.4463\right)\mathrm{nFAC}\sqrt{\frac{{\mathrm{nFvD}}_{0}}{\mathrm{RT}}}$$

(7) $$I\%=\left(\frac{I_{\mathrm{modified}}-I_{\mathrm{bare}}}{I_{\mathrm{bare}}}\right)\cdot 100$$

in which, I_modified_ corresponds to the faradic current value obtained using CNTs-modified SPEs and I_bare_ to the faradic current value obtained with bare SPEs. 

## 3. Results and Discussion

### 3.1. Morphological Characterization of Carbon Nanomaterials (CNMs) Modified-Platforms

**Figure 1:**
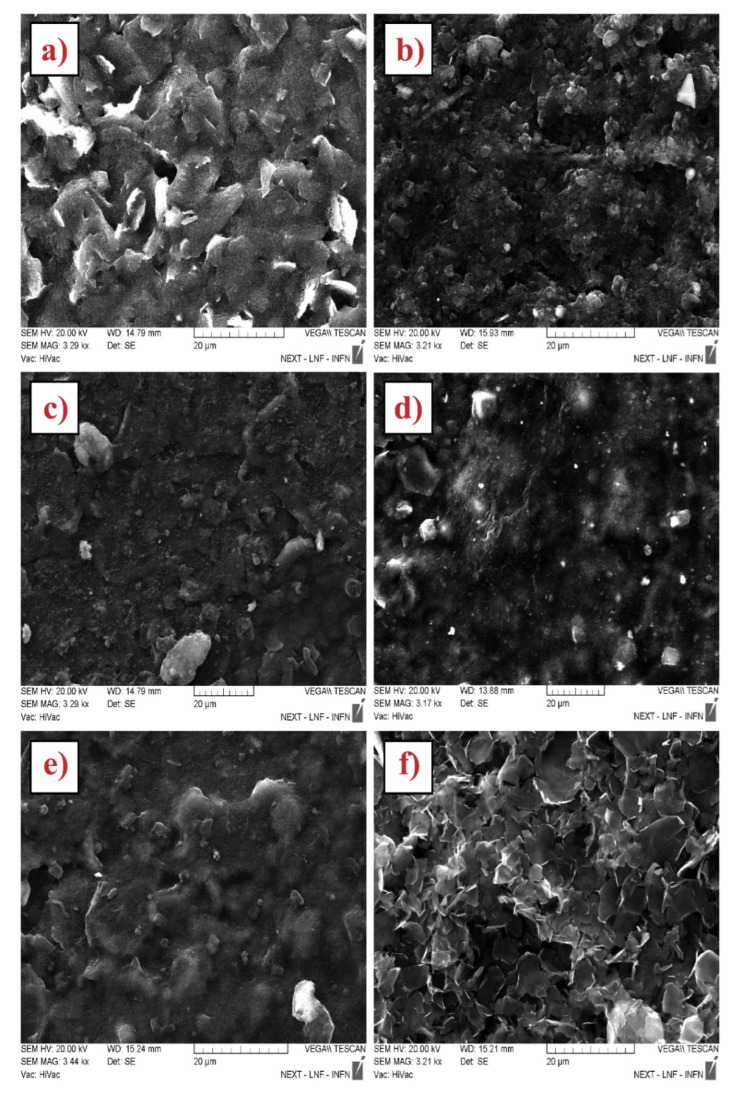
SEM characterization: (**a**) Bare SPE, (**b**) Bare MWNT, (**c**) CO_2_H-MWNT-SPE, (**d**) NH_2_-MWNT-SPE, (**e**) OH-MWNT, and (**f**) GNP-modified SPE.

**Figure 2:**
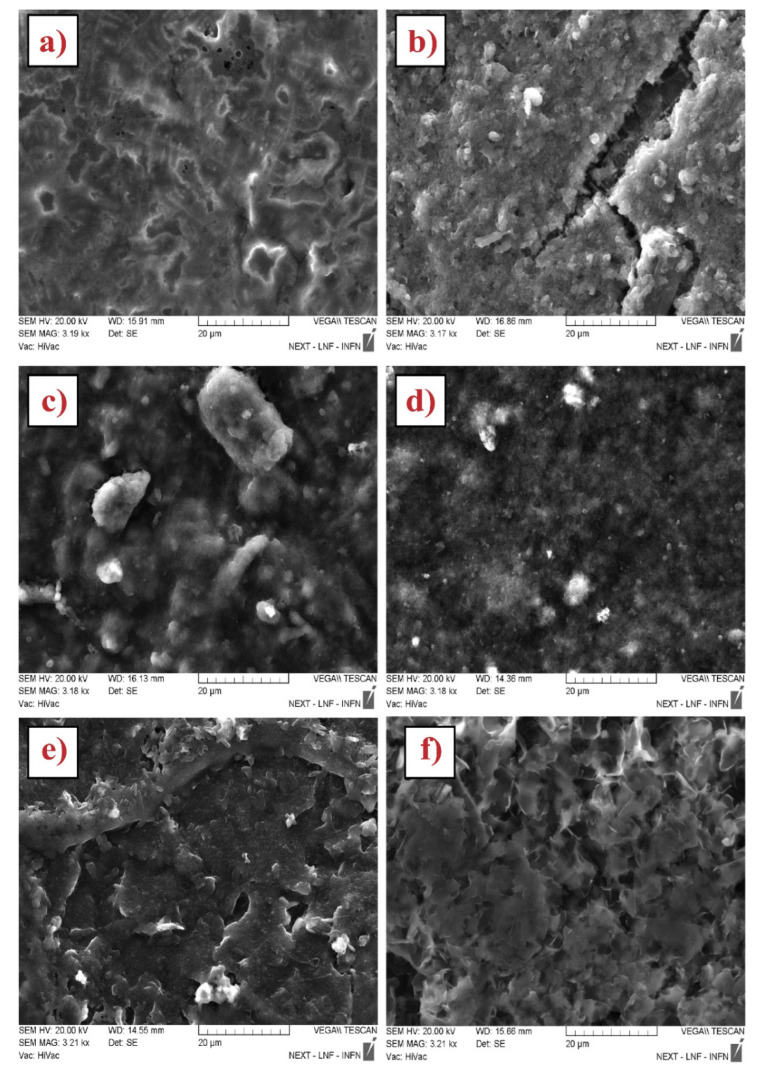
SEM characterization of uricase biosensors based on: (**a**) Bare SPE, (**b**) Bare MWNT, (**c**) CO_2_H-MWNT-SPE, (**d**) NH_2_-MWNT-SPE, (**e**) OH-MWNT, and (**f**) GNP-modified SPE.

To investigate the surface modification of working electrodes before and after CNMs drop casting, an SEM observation was carried out. A representative micrograph for each platform is reported in Figure 1. Analyzing the surface of unmodified SPE (bare SPE), reported in Figure 1a, graphite particles emerged from the polymer matrix used for printing purposes. The comparison with the surface of CNMs-modified SPEs highlights that the nanostructures retraced the underlying graphite particles and consequently increased the surface area of working electrodes: the nanomaterials are not coated by the polymer matrix of the conductive inks, and this factor helps dramatically to enhance the surface area. The WE modification by means of Uricase severely changes the electrodic surface, is shown in Figure 2.

The uricase adheres homogeneously to the carbonaceous structures: a thin layer of enzyme coats the graphite (bare SPE, Figure 2a) and carbon nanostructure (Figure 2b–f), clearly retracing the underlying structure. The homogeneous and thin coating of the nanostructures is important to ensure a good sensing behavior. In fact, the high surface area obtained by the deposition of nanostructures is now available to detect the electrochemical response selectively by means of an enzyme. The thin enzyme coating guarantees the electrodic area does not decrease. Moreover, the homogeneous coating allows all enzymes to be close to the nanostructure and carry out good selectivity conjugates with high electrochemical properties.

### 3.2. Electrochemical Characterization of CNMs Modified-Platforms

**Figure 3:**
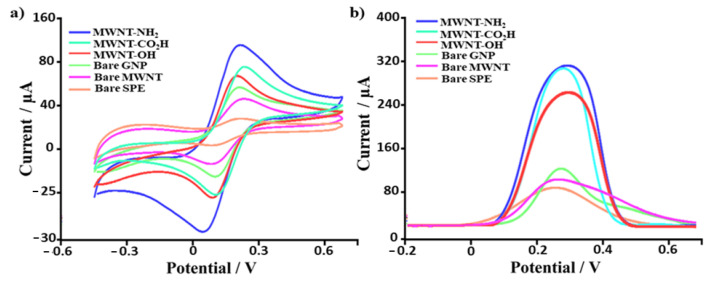
(**a**) Cyclic and (**b**) Square Wave voltammogram traces recorded using bare and nanomaterial-modified SPEs (GNP, Bare MWNT, MWNT-OH, MWNT-CO_2_H and MWNT-NH_2_) in the presence of 1 mM potassium hexacyanoferrate (III) + 0.1 M KCl, pH 7.4. In (**a**), all traces were recorded at a scan rate of 30 mV s^−1^; in (**b**), the amplitude and frequency are equal to 0.05 V and 5.0 Hz, respectively.

To ascertain the electrochemical properties that carbon nanomaterials (CNMs) confer to screen-printed electrodes, CV (Figure 3a) and SWV (Figure 3b) analyses were performed using [Fe(CN)_6_]^3−/4−^ as an electroactive probe. 

By analyzing the voltammograms reported in Figure 3, it can be readily observed that the presence of nanomaterial-based functionalization produces a dramatic improvement in the magnitude of the voltammetric peak height, reported as the percentage increase (% increase) of the anodic and cathodic peak current in Table 2 (from 2 to 7-fold increase in the registered anodic and cathodic peak current). This electrocatalytic response, which formally is a decrease in the overpotential and an increase in the peak current, can be ascribed to the increase of the electrodic surface area originated by nanomaterial modification. Indeed, from the examination of the Randles–Sevcik equation (Equation (6)), one can easily observe that if everything is kept unchanged, with only the electrode area left free to change, the corresponding voltammetric peak height will proportionally increase. This physical post-printing modification is perceived in electrochemical measurements by increasing the voltammetric peak height. This, in turn, allows the measurement of a lower concentration of the target analyte, giving rise to a lower LOD compared to the unmodified SPE (Table 2) and, consequently, an increase in the slope of a plot of peak current against concentration is observed (sensitivity). Reproducibility, peak-to-peak separation (index of ideal behavior of electron transfer process), electron transfer rate constant, and the ratio of the anodic and cathodic peak current intensity were also analyzed. The corresponding results are summarized in Table 2. All the examined analytical parameters confirm the suitability of bare and functionalized CNMs in the electrodic surface area modification, giving rise to a massive improvement in the electrochemical properties of the platforms.

The analysis of the above-reported parameters shows that the best results in terms of I% were obtained with aminic (NH_2_) functionalized MWNTs (7 and 5-fold increase for anodic and cathodic peak current, respectively). Whereas the most promising results in terms of repeatability and reproducibility (RSD%) were obtained for carboxylic (CO_2_H) functionalized MWNTs (3%, significantly lower than bare electrodes reproducibility, which is equal to 11%). The same nanomaterial-modified SPEs, thus NH_2_ and CO_2_H functionalized MWNT, present the best results in terms of sensitivity and limit of detection (LOD). These improvements can be ascribed to an increase in the electrodic surface area due to the presence of carbon-based nanomaterials and an enhanced electron transfer rate constants (k^0^). Moreover, unfunctionalized MWNTs (bare) showed the smallest peak-to-peak separation (ΔE); thus, an ideal reversible electrochemical behavior was observed for these nanoengineered platforms. Finally, the GNP-modified platform showed the best result concerning the ratio of the anodic and cathodic peak current intensity.

All the reported electrochemical parameters undoubtedly confirm how these well-defined nano-carbonaceous materials possess the required surface structure and electronic properties to support rapid electron transfer, good reproducibility, and sensitivity for electrochemical biosensor implementation.

### 3.3. Electrochemical Performances of CNMs-Based Uric Acid Biosensor

All CNMs-based platforms, once morphologically and electrochemically characterized, were applied as sensing devices for the enzymatic biosensor fabrication. The anodic current recorded in the chronoamperometric traces is due to the oxidation of hydrogen peroxide (H_2_O_2_) obtained as a secondary product of the enzymatic conversion of uric acid into allantoin by uricase. The H_2_O_2_ concentration will be proportional to the UA concentration, making possible the amperometric quantification of the target analyte. 

**Figure 4:**
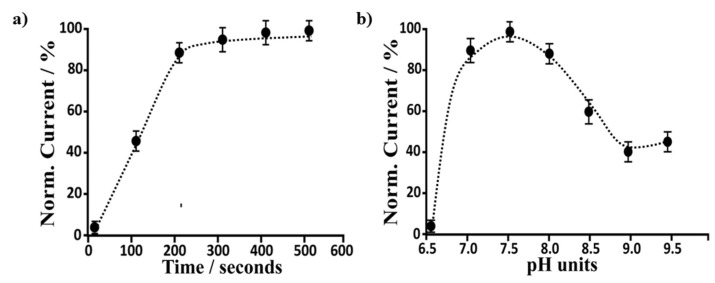
(**a**) Time response and (**b**) working pH values optimization were reported. The results were obtained by analyzing amperometrically bare SPEs in the presence of 1 μM UA (in a buffer solution, PBS).

The time response and pH values of electrolytes have a great influence on biosensor activity. Therefore, these two conditions have been carefully optimized. The time is highly suitable for biosensor response. In particular, the current recorded using the bare SPEs-based uricase biosensor at a fixed concentration of UA (1 nM) was plotted against time, and the obtained curve is reported in Figure 4a. The time response selected, where the current values are approximately constant, was 300 s. Once the response time was optimized, the optimization of the uricase-based biosensors was carried out at a continuous uric acid concentration (1 nM) using bare SPEs-based biosensors. Figure 4b showed the effect of varied pH values in a fixed concentration of UA (i.e., 1 nM). Subsequently, the optimal pH for the enzymatic reaction was set up by testing values in the pH range of 6.5–9.5. The recorded current increased when increasing the pH from 6.5 to 7.5. When the pH value was increased further, up to 8.5, the peak current began to decrease. Therefore, pH 7.5 was selected as the best pH condition.

**Figure 5:**
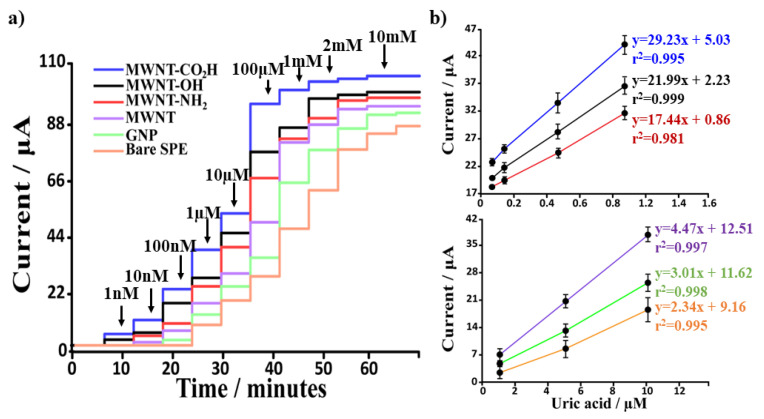
(**a**) Amperometric traces recorded, using a potential 0.05 V and the corresponding (**b**) calibration curves obtained using bare and nanomaterial-modified biosensor (GNP, Bare MWNT, MWNT-OH, MWNT-CO_2_H and MWNT-NH_2_) in the presence of an increasing concentration of uric acid (addition method to have a concentration from 0.1 nM to10 mM).

All the nanomaterial-based biosensors were separately tested, and the overall outcomes are summarized in Figure 5 (original traces are reported in Figure S1 in Supplementary Materails). Here, the anodic current values attained by the addition method are reported (Figure 5a). This experiment has been realized, including all the different platforms and the deriving analytical parameters (LOD, K_m_ and linear range) compared with the uric acid biosensor based on the bare electrode (Table 3). As one can easily notice, the use of nanomaterial-based platforms positively affects the performance of the biosensors (increased anodic current registered), significantly lowering the LOD and extending the linear range. These properties allow us to detect and quantify lower uric acid concentrations (with respect to biosensors based on bare SPE), making the nanoengineered platforms more sensitive and wide-range applicable.

**Figure 6:**
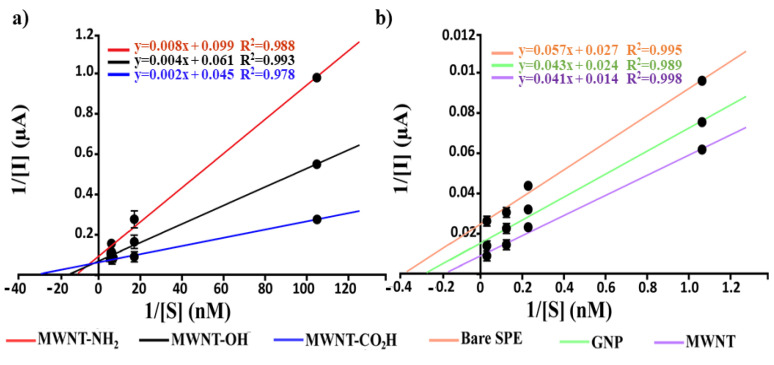
(**a**) Lineweaver–Burk plots obtained using (**a**) functionalized CNMs (MWNT-OH, -CO_2_H, and NH_2_) and (**b**) GNP, Bare MWNT and bare SPE based biosensors in the presence of an increasing concentration of uric acid.

The current recorded using functionalized MWNT-based biosensors (CO_2_H, OH, NH_2_) was linear to a UA concentration within the range of 0.1 nM–1 μM for MWNT-modified SPEs. In contrast, a 1 nM–1 mM linear range was obtained for GNP and bare SPEs-based UA biosensors. The linear regression equation was y = a + bx, where y was the current (μA, sampled at 270 s), and x was the UA concentration (nM) with the relative R squared correlated parameter is reported in Figure 5b. According to the calibration curve of current and concentration, the sensitivity and the limit of detection (LOD, calculated using Equation (3)) was reported in Table 3. In particular, the standard deviation (σ) necessary for LOD calculation was obtained by analyzing the amperometric currents recorded for blank solution (zero concentration of UA). Finally, the kinetics of the immobilized enzyme (uricase) were calculated (Figure 6), as already shown in our previous works [23,24], using the apparent Michaelis–Menten constant (K_m_) by the linearization of Lineweaver–Burk expressed by Equation (8):

(8) $$\frac{1}{I}=\frac{1}{I_{\max}}+\frac{k_{m}^{\mathrm{app}}}{I_{\max}\left[S\right]}\;$$

where [S], which is the concentration of the substrate, I is the cathodic current recorded applying 0.05 V potential, $k_{m}^{\mathrm{app}}$ is the apparent Michaelis–Menten constant for the enzymatic reaction, and I_max_ is the steady-state current. The apparent Michaelis–Menten constant values provide important information regarding the interaction between the enzymatic structure and the target analyte. In particular, the lower the K_m_ value, the higher the substrate affinity. Analyzing Table 3, one can notice that functionalized CNMs-based biosensors (-CO_2_H, -OH, -NH_2_) show better affinity than the biosensors based on unfunctionalized CNMs (bare MWNTs and bare GNPs) and bar electrodes. This can be ascribed to the presence of functional groups, which not only electrostatically stabilize the enzyme, thus not perturbing the catalytic site and its accessibility to UA, but also improve the electron transfer process at the electrode surface as detailed above.

### 3.4. Stability, Reproducibility and Specificity of CNMs-Based Uric Acid Biosensor

**Figure 7:**
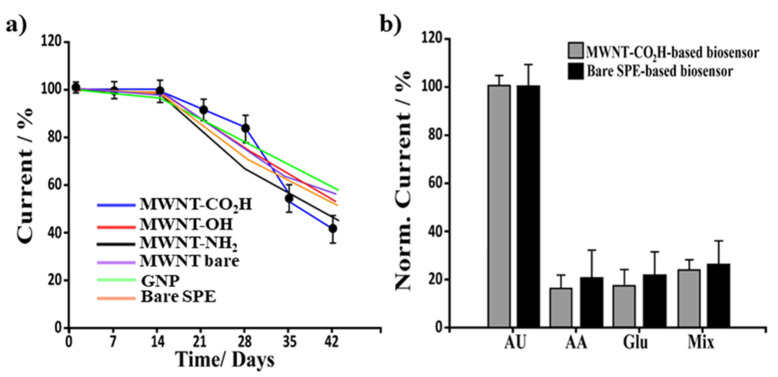
Stability and selectivity of CNMs-based UA biosensors. (**a**) Stability study. All the biosensors were tested after several days of storage at 4 °C in a humid chamber, and amperometrically analyzed at the indicated times with 10 µM UA concentration. (**b**) Selectivity study. Effect of the presence of the AA, Glu, and their mix on the biosensor responses obtained for 10 µM UA solution.

The stability of the CNMs-based biosensors was examined by repeating the measurements of 10 μM UA periodically over six weeks. The investigation was realized for all the different platforms stored in a humid chamber at 4 °C without using any preservatives. The results, reported in Figure 7a, showed an almost constant response for up to fifteen days after their preparation (similar results in terms of registered faradic current). After this period, a significant drop in the electrochemical performances were found, thus indicating a time-dependence of enzymatic activity. Moreover, the biosensor showed an almost identical response (the signal loss is <5%), up to 10 successive measurements with relative standard deviation (RSD) ranging from 5% (-CO_2_H, -OH, -NH_2_, Bare MWNT, GNP) to 16% (bare SPEs-based biosensors), thus indicating good applicability. 

The repeatability (Table S1 in Supplementary Materails).) and the reproducibility of the biosensors (Table S2 in Supplementary Materails).) was investigated from the response to 10 μM UA at three electrodes fabricated by the uniform procedure. The functionalized-based biosensors (CO_2_H, OH, and NH_2_) showed high reproducibility with an RSD of 6, 4, and 5%, respectively. Whereas bare MWNT, GNP, and bare electrodes-based UA biosensors showed reproducibility of 10, 9, and 15%, respectively. 

The specificity of the biosensor towards UA was investigated by studying some of the interferents commonly found in real samples, such as glucose (Glu) and ascorbic acid (AA). In this study, biosensors based on carboxylic functionalized CNMs have been tested using a fixed concentration (10 μM) of Glu, AA, and a mix of them (1:1 *v/v* ratio). The results, comparable to those of SPE-based biosensors (Figure 7b), showed that the fabricated biosensor had a strong anti-interference ability.

### 3.5. Preliminary Application of CNMs-Based Uric Acid Biosensor in Urine Samples

**Figure 8:**
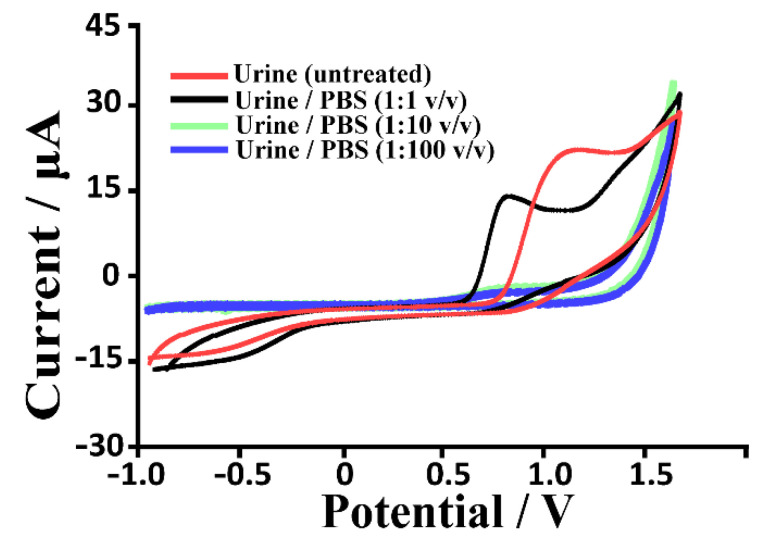
Cyclic voltammograms traces recorded using MWNT-CO_2_H-modified SPEs in diverse urine/PBS dilution ratio (untreated, 1:1, 1:10 and 1:100 *v/v*). all traces are recorded at a scan rate of 30 mV s^−1^.

To test the ability of the developed biosensors to be used in a real matrix, their application on human urine samples was investigated. Initially, different urine/PB dilutions ratios (untreated, 1:1, 1:10 and 1:100 *v/v*) were studied. In particular, CV and carboxylic functionalized MWNT-based biosensors were used to verify the presence of any electrochemical interferences correlated to the urine matrix components. From the voltammograms depicted below (Figure 8), it is possible to observe a peak current (between 0.6 and 0.9 V, ascribable to the presence of endogenous UA and AA), which gradually disappears with increasing dilutions. However, the potential presence of electroactive endogenous species and the problems associated with them were easily overcome by diluting the samples (see dilution urine/PBS 1:100 *v/v*) and using PB as a diffusion mediator (fixed potential measurements, 0.05 V). The standard addition method was used to study the analytical performances of our nanomodifed platforms in spiked urine samples, and the overall outcomes are summarized in Table 4. Moreover, a recovery study using CO_2_H-MWNT and bare SPE-based biosensors, and analyzing a series of known UA concentrations (0.1, 10, 50 and 100 μM) (Table 5, estimating % recovery value), was carried out following the procedure explained by Verma et al. (2019) in their work [58]. In particular, the % recovery was calculated following Equation (9).

(9) $$\%\mathrm{Recovery}=\frac{\left(C_{T}-C_{0}\right)}{C_{S}}\times 100$$

where *C_T_* is the total concentration of UA in the spiked urine samples estimated from the calibration plot, *C*_0_ is the concentration of UA in the unspiked sample (1.2 μM calculated according to the response current with the standard calibration curve), and *C_S_* is the concentration of analyte spiked into the sample. The % recovery values for the spiked samples were 89.0 to 95.3% and 78.0 to 89.2% for CO_2_H-MWNT and bare SPE-based biosensors, respectively. Therefore, a negligible matrix effect and good efficiency of the developed biosensors were verified towards the analysis of AU in clinical detection. Normal excretion of uric acid in urine samples ranges from 250 to 750 milligrams per day (concentration between 250 and 750 mg/L if one litre of urine is produced per day), an amount easily detectable with our sensor. Moreover, the great sensitivity of this biosensor allows us to significantly dilute complex real matrices (i.e., diabetic and patients undergoing chemotherapy therapies, etc.) to further minimize the interfering effect of other components.

## 4. Conclusions

In this paper, an in-depth, electrochemical and morphological characterization of CNMs-modified SPEs has been reported. In particular, CV, allowing us to understand and explain the chemistry related to the different functionalization of the CNMs employed in SPE construction, undertook a quantitative characterization study of electron transfer processes at the diverse CNMs-modified interfaces. Successively, CNMs-SPEs were used for uric acid determination, immobilizing uricase on CNMs-modified platforms using Prussian Blue as an electrochemical mediator. The amperometric response of the uricase-CNMs-PB biosensors was optimized in terms of pH, time analysis, and applied potential, reaching a detection limit of nanomolar level. The results revealed that the prepared biosensor could successfully detect UA at a wide concentration range of 0.1 nM–100 μM with a very low detection limit (CO_2_H) of 0.5 nM, and a lower k_m_ value of 0.04 μM with respect to bare electrode-based biosensors (LOD 280 nM and k_m_ 4 mΜ). At the same time, it exhibited good stability and specificity towards UA in the presence of interferent analytes and good performances in the human urine sample, which confirms its high potential for the efficient detection of UA that can be extended for use in clinical settings.

## Figures and Tables

**Table 1:** Comparison of different UA detection methods.

Method			LOD [nM]	Ref.
Biosensors Detection Methods	Luminescent	Colorimetric	20–50	[35,36]
		Fluorescence	200	[35,36]
		Chemlum.	(1–3) × 10^3^	[37,38]
	Amperometric		5–100	[32,44]
	Potentiometric		(2–5) × 10^3^	[45]
High-Performance Liquid Chromatography			10–100	[42,43]
Capillary Electrophoresis			(2–3) × 10^3^	[40,41]
Electrochemical Methods	Voltammetry	DPV	10–100	[46,47]
		SWV	1–100	[48,49]

**Table 2:** Cathodic and anodic peak current intensity, ratio of the anodic and cathodic peak current intensity, electron transfer rate constant (k^0^), peak-to-peak separation (ΔV), LOD, sensitivity, reproducibility (RSD%), and percentage increase estimated for bare and nanomaterial-modified SPEs have been reported. All analytical parameters are obtained from cyclic voltammogram traces.

	Bare Electrode	Bare MWNT	-CO_2_H MWNT	-OH MWNT	-NH_2_ MWNT	Bare GNP
Fe(CN)_6_^4−/3−^						
|Iap| [µA]	17 ± 2	46 ± 3	93 ± 3	75 ± 2	147 ± 6	64 ± 4
|Icp| [µA]	10 ± 1	38 ± 2	76 ± 2	62 ± 3	115 ± 5	70 ± 4
|Ipa|/|Ipc|	1.7	1.2	1.2	1.2	1.3	0.91
*k*^0^ [cm/s]	/	3.3 × 10^−3^	1.7 × 10^−3^	2.2 × 10^−3^	5.6 × 10^−3^	2.8 × 10^−3^
ΔE [mV]	380	77	92	85	70	80
LOD [μM]	34.8	2.2	1.2	1.1	0.9	3.4
Sensitivity [mA/M cm^2^]	11.6	6.4	4.5	5.5	5.7	6.5
Reproducibility |Iap|	11	6	3	3	4	6
Reproducibility |Icp|	10	5	3	5	4	5
% Increase |I_pa_|	/	177	460	352	764	276
% Increase |I_pc_|	/	274	668	516	576	312

**Table 3:** LOD, sensitivity, and Km (Michaelis–Menten constant) estimation for bare and CNMs-modified biosensors. These analytical parameters were obtained from chronoamperometric traces (E, time interval and time run respectively equal to 0.05 V, 0.01 s, and 300.0 s).

	Bare Electrode	Bare MWNT	-CO_2_H MWNT	-OHMWNT	-NH_2_ MWNT	Bare GNP
LOD [nM]	280	74	0.5	0.9	2.1	98
Sensitivity [μA μM^−1^ cm^−2]^	33	64	418	314	249	43
K_m_ [nM]	3.0	1.8	0.04	0.07	0.08	2.1

**Table 4:** LOD, sensitivity and reproducibility estimated for bare and CNMs-modified biosensor in urine matrix. These analytical parameters are obtained from chronoamperometric traces (E, time interval and time run respectively equal to 0.05 V, 0.01 s, and 300.0 s).

	Bare Electrode	Bare MWNT	-CO_2_H MWNT	-OH MWNT	-NH_2_ MWNT	Bare GNP
LOD [nM]	1400	213	2.2	3.8	8.3	420
Sensitivity [μA mM^−1^ cm^−2^]	13	64	418	314	249	43
RSD%	17	10	9	9	10	11

**Table 5:** Determination of UA concentrations in spiked urine samples.

	Spiked UA Concentration *C_S_* (μM)	Recovered UA Concentration (*C* − *C*_0_) (μM)	Recovery% (n = 6)	RSD% (n = 6)
**CO_2_H**	10	8.9	89.0	4
	50	46.8	93.6	3
	100	95.3	95.3	3
**Bare SPE**	10	7.8	78.0	14
	50	44.4	88.9	12
	100	89.2	89.2	11

## Supplementary Materials

The following are available online at https://www.mdpi.com/article/10.3390/bios12010002/s1, Figure S1: The original experiment results exploited to create Figure 5a, Table S1: Repeatibility of CNMs-based UA-biosensors response, Table S2: Reproducibility of CNMs-based UA-biosensors response.
