# Supplementary material for: Powerful Electron-Transfer Screen-Printed Platforms as Biosensing Tools: The Case of Uric Acid Biosensor
Source: Biosensors (Basel). 2021 Dec 21;12(1):2. doi: 10.3390/bios12010002 (PMC8773917; doi:10.3390/bios12010002)
Supplement: Supplementary file 1 [file biosensors-12-00002-s001.zip › biosensors-1480953-supplementary.pdf]

biosensors

MDPI

Supplementary Materials

Powerful Electron-Transfer Screen-Printed Platforms as Biosensing Tools: The Case of Uric Acid Biosensor

Rocco Cancelliere [1], Alessio Di Tinno [1], Antonino Cataldo [2], Stefano Bellucci [2,*] and Laura Micheli [1,*]

[1] Department of Chemical Sciences and Technologies, University of Rome Tor Vergata, Via della Ricerca Scientifica 1, 00133 Roma, Italy; rocco.cancelliere@uniroma2.it (R.C.); alessio.ditinno@uniroma2.it (A.D.T.)
[2] INFN-Laboratori Nazionali di Frascati, Via E. Fermi 54, 00044 Frascati, Italy; antonino.cataldo@lnf.infn.it
* Correspondence: bellucci@lnf.infn.it (S.B.); laura.micheli@uniroma2.it (L.M.)

Repeatability and Reproducibility (Statistical Tests)

The repeatability was studied for all concentrations using each nanomodified platform (n = 3, each concentration). Whereas, the reproducibility was checked using five equally prepared electrodes for each CNMs-based platform by analysing three UA concentrations 1, 10 (reported in the manuscript) and 100 μM. The repeatability and reproducibility statistical tests for three different concentrations (1, 10, 100 μM) are reported in the following tables.

Table S1. Repeatability of CNMs-based UA-biosensors response.

	Repeatability Current Response (μA)					
**1 μM**	**CO_2H**	**NH_2**	**OH**	**MWNT**	**GNP**	**Bare SPE**
	36.5	21.9	25.4	18.9	16.3	14.3
	37.7	22.7	24.3	17.8	14.7	16.1
	38.8	22.5	24.7	17.4	15.4	13.8
**RSD%**	**3**	**2**	**2**	**4**	**5**	**8**
**10 μM**	51.6	38.5	41.7	33.7	28.6	18.6
	51.5	37.3	42.5	34.1	28.4	22.2
	50.1	38.5	43.6	31.7	31.2	17.9
**RSD%**	**2**	**2**	**2**	**4**	**5**	**12**
**100 μM**	92.2	77.6	64.7	52.5	38.3	33.3
	91.1	78.3	62.2	49.7	36.1	24.5
	94.7	80.1	63.6	51.2	34.7	30.2
**RSD%**	**2**	**2**	**2**	**3**	**5**	**14**

Table S2. Reproducibility of CNMs-based UA-biosensors response.

	**Reproducibility Current Response (µA)**					
**1 µM**	**CO_2H**	**NH_2**	**OH**	**MWNT**	**GNP**	**Bare SPE**
	33.6	25.4	21.5	20.9	15.4	13.1
	38.1	24.2	22.6	17.8	14.9	16.4
	37.8	23.9	22.5	18.4	15.4	13.8
	35.5	22.1	20.7	15.6	16.6	15.9
	33.7	24.5	20.8	18.1	17.8	17.7
**RSD%**	**6**	**5**	**4**	**12**	**7**	**12**
**10 µM**	51.6	39.2	43.4	35.3	29.5	15.6
	52.3	36.7	44.5	31.2	28.4	21.2
	47.5	36.4	45.6	39.3	34.2	21.6
	46.8	36.8	41.2	32.6	26.9	17.5
	45.7	40.5	46.1	31.1	31.2	22.2
**RSD%**	**6**	**5**	**4**	**10**	**9**	**15**
**100 µM**	89.9	75.5	61.4	47.5	31.3	32.3
	89.1	78.3	62.2	56.7	40.1	27.5
	95.2	80.6	60.6	49.3	38.1	35.2
	97.7	73.3	64.5	52.3	37.6	26.8
	96.2	76.6	66.2	54.1	42.9	25.9
**RSD%**	**4**	**4**	**4**	**7**	**11**	**14**

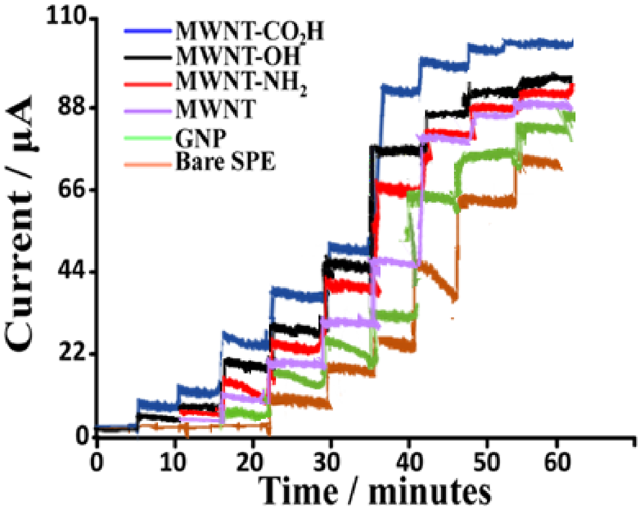

Figure S1. The original experiment results exploited to create Figure 5a.
